# Interobserver Variability in Cardiovascular FDG PET/CT Analysis in Large Vessel Vasculitis

**DOI:** 10.5334/gh.1433

**Published:** 2025-05-28

**Authors:** Redemptar Kimeu, Anoop Shah, Samuel Gitau, Gemina Doolub, Jeilan Mohamed

**Affiliations:** 1 The Nairobi Hospital, Nairobi, KE; 2 London School of Hygiene and Tropical Medicine, UK; 3 Agakhan university hospital, Nairobi, KE; 4 Translational Health Sciences, University of Bristol, UK; 5 Agakhan University Hospital, Nairobi, KE

**Keywords:** Vasculitis, FGD/CT PET, Large vessel vasculitis, interobserver variability, standardized uptake values

## Abstract

**Introduction:**

PET/CT has a synergistic value for optimal diagnosis, disease activity monitoring, and evaluation of damage progression in large vessel vasculitis. The use of standardized uptake values (SUV) as a measurement of relative tissue uptake facilitates comparisons between patients, and has been suggested as a basis for diagnosis. The SUVmean and SUVmax reproducibility in vascular structures is not widely studied.

**Objective:**

The objective of this study was to evaluate the inter-observer variability of both qualitative visual grading of aortic ^18^F-FDG uptake and the quantitative aortic mean and maximum SUVs in these patients with mild to moderate covid-19 infection who underwent multimodality cardiac imaging within the COSMIC-19 trial.

**Study design:**

This is a sub-study of the COSMIC-19 trial. 30 patients were subjected to a combined Computed Tomography Coronary Angiogram and ^18^F-FDG PET/CT, followed by cardiac magnetic resonance. Two independent observers measured the Standardized uptake values in five regions of interest at each aortic segment. These were performed sequentially along the length of the aorta every 5 mm on the axial slices. The maximum and mean standard uptake values were measured.

**Results:**

Qualitative assessment showed excellent agreement between observer x and y for the ascending aorta and aortic arch regions with the kappa coefficients for the inter observer agreement of 0.92 (95% CI:0.78–1.0) and 0.91 (95% CI:0.74–1.0) respectively. Quantitative assessment showed a very high positive correlation between the two observers for each of the regions measured for SUVmean as follows; ascending aorta r = 0.96 (p < 0.001), Aortic arch r = 0.90 (p < 0.001) and descending Aorta r = 0.91 (p < 0.001). The correlation coefficients for the SUVmax were substantially strong.

**Conclusion:**

This study shows an excellent inter-observer reproducibility for both qualitative and quantitative SUVmean vascular ^18^F-FDG measurements in patients with COVID-19 large vessel vasculitis. Quantitative SUVmax demonstrated substantially strong interobserver reproducibility.

## Introduction

Integrated positron emission tomography/computed tomography (PET/CT) is the first non-invasive technique to allow precise localization of abnormal isotope uptake in inflamed arterial walls (1,2). The utility of 18F-fluorodeoxyglucose-PET (^18^F-FDG PET) as a diagnostic tool in the investigation of vascular disease is based on its ability to detect enhanced glucose uptake from high glycolytic activity of these inflammatory cells (3). PET/CT has a synergistic value for optimal diagnosis, disease activity monitoring, and evaluation of damage progression in large vessel vasculitis (LVV) (4). The sensitivity of PET/CT in identifying findings consistent with LVV has been reported to range from 77–92%, and the specificity from 89–100% (5).

Currently, PET/CT vasculitis evaluation is derived from recommendations published in 2018 on the diagnosis and follow-up of patients with suspected or diagnosed with LVV (6). Several PET interpretation criteria have been proposed, with the most common over the last 15 years being the use of a visual grading scale (vascular to liver uptake). This is a standardized four-point scale grading system: 0 = no uptake (≤mediastinum); 1 = low-grade uptake (<liver); 2 = intermediate-grade uptake (= liver); 3 = high-grade uptake (>liver). In this system, grade 2 is considered possibly indicative, and grade 3 is considered positive for active LVV (7,8).

The recent interest in using ^18^F-FDG PET in assessing disease activity and monitoring early response to therapy has made quantitative measurements much more important (9). PET semi-quantitative analysis includes target-to-background ratio (TBR), which evaluates the normalization of the arterial wall standard uptake values to the background activity of venous blood pool, hence providing a good reference for assessing vascular inflammation. Grading of arterial inflammation against the liver background is also an established method (6).

The most common parameter used to measure tracer accumulation in PET studies is the standardized uptake value (SUV). The SUV is a semi-quantitative measure of normalized radioactivity concentration in PET images. The standardized uptake value (SUV) is commonly used as a relative measure of ^18^F-FDG PET uptake to compensate for the amount of ^18^F-FDG injected and the weight of the patient. The use of SUVs as a measurement of relative tissue uptake facilitates comparisons between patients and has been suggested as a basis for diagnosis (10).

There are two common ways of reporting SUV: the mean SUV (SUVmean) or the maximum SUV (SUVmax) of all voxels within the region of interest (ROI). SUVmean incorporates information from multiple voxels, making it less sensitive to image noise. However, measured SUVmean will vary depending on which voxels are included in the average, so it is sensitive to ROI definition and is subject to intra- and interobserver variability (9). SUVmax has excellent reproducibility for solid structures such as the liver and oncologic masses (11). The SUVmax reproducibility in vascular structures is not widely studied. Likewise, the interobserver variability of quantitative SUV assessment in vascular structures, such as the aorta of patients with and without vasculitis, has not previously been studied in our region.

The objective of this study was to evaluate the interobserver variability of both qualitative visual grading of aortic ^18^F-FDG uptake (gold standard) and the quantitative aortic mean and maximum SUVs in 29 patients with mild to moderate COVID-19 infection and one control who underwent multimodality cardiac imaging within the Cardiovascular Mechanisms in COVID-19 (COSMIC-19) trial (12).

## Materials and Methods

### Study design

**Figure 1:**
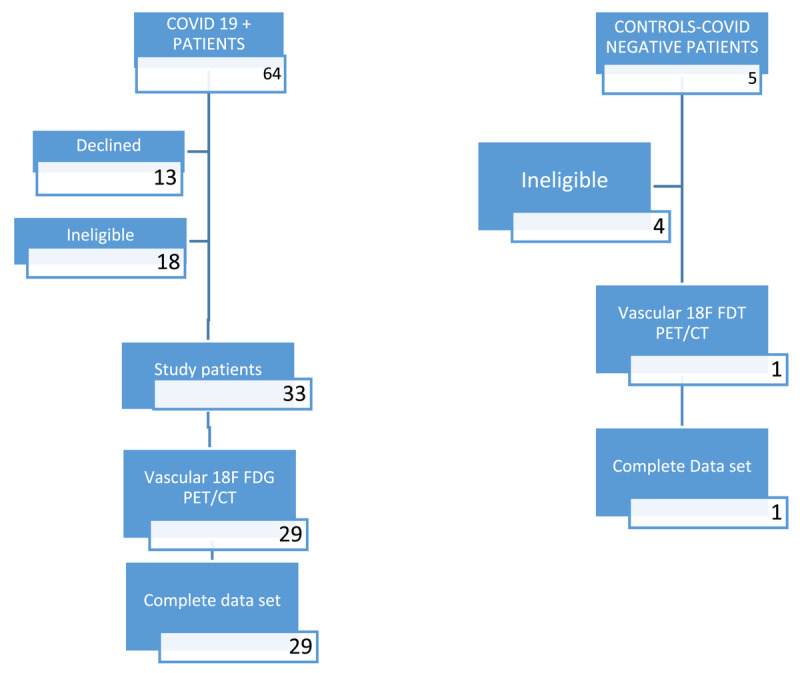
Recruitment of study patients and controls. Vascular 18-F FDG PET/CT was completed in 30 patients; 29 study patients and one control patient. The 30 complete data sets were eligible for inter-observer variability assessment.

This is a sub-study of the COSMIC-19 trial (12), a single-centre exploratory observational study in patients with COVID-19 positive status on polymerase chain reaction testing receiving care at the Aga Khan University Hospital in Nairobi, Kenya. Consecutive patients presenting with acute COVID-19 were prospectively recruited during hospital admission in this cross-sectional study. A total of 64 patients were recruited, and only 33 met the inclusion criteria. Five control patients were recruited (Figure 1). Following informed consent, blood draws were taken for high-sensitivity cardiac troponin, N-terminal pro B-type natriuretic peptide and high-sensitivity C-reactive protein. Patients then underwent multimodality imaging: simultaneous CTCA and thoracic ^18^F-FDG PET/CT (GE Discovery MI series PET/CT scanner) within two weeks of presentation to maximise sensitivity of the scan, followed by CMR (Ingenia, Philips Healthcare, as described previously (CMR) (Complete protocol)) (13). The study measures the mean and maximum SUVs in five ROIs at each aortic segment: the ascending aorta (aa), arch of the aorta (arch) and the descending aorta (da).

### Selection criteria for COSMIC-19 trial

Inclusion criteria captured patients diagnosed with COVID-19 aged over 18 years who tested positive on PCR testing, ideally within two weeks of a positive test. Patients were excluded based on the following conditions: (1) prior diagnosis of myocardial infarction, coronary revascularization or cardiac surgery; (2) requiring invasive or non-invasive ventilation; (3) inability to undergo CT or CMR scanning; (4) severe renal failure (estimated glomerular filtration rate < 30 mL/min); (5) major allergy to iodinated contrast media/gadolinium; (6) pregnancy or breastfeeding; (7) inability to give informed consent; (8) contraindication to imaging (e.g., metal fragments in the eye). Similarly, exclusion criteria applied to patients over the age of 18 years chosen as controls and testing negative for COVID-19 (13).

All the patients who underwent multimodality cardiac imaging were included in this sub-study. Participants underwent ^18^F-FDG PET imaging after a low-carbohydrate diet for 24 hours, followed by a 12-hour fast to suppress physiologic FDG uptake by normal myocytes (14). The PET imaging was performed 60 minutes after administration of 10 mCi of ^18^F-FDG using a 64-slice General Electric (GE) Discovery MI series PET/CT scanner. The carotid arteries were the superior aspect of the imaging, and the entire thoracic aorta and heart were covered using different bed positions. Corresponding low-dose CT images were obtained immediately after PET scan acquisition for correction and anatomical registration (13).

**Figure 2:**
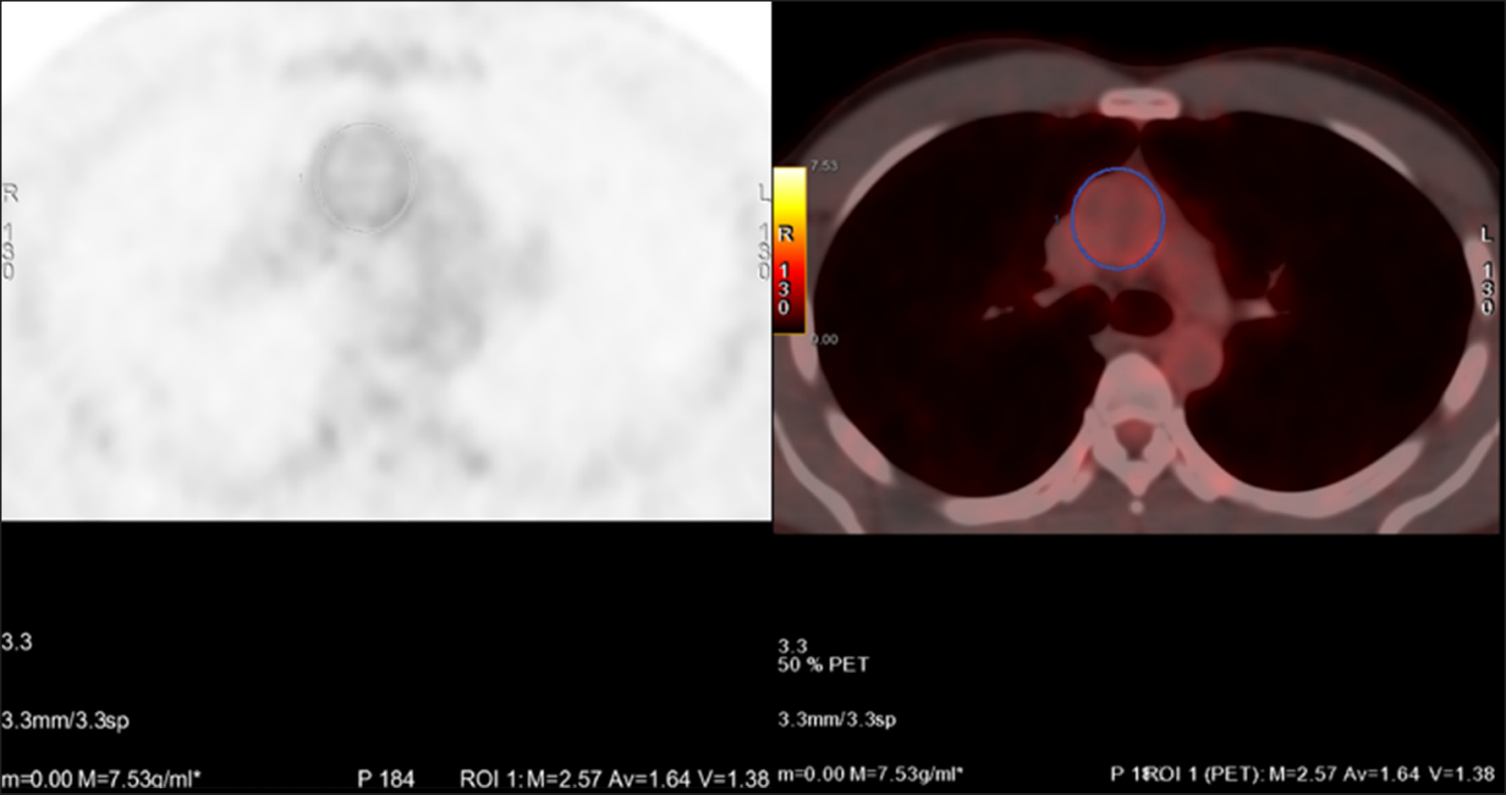
Standard uptake values measurements at various levels of axial slices of the ascending aorta. The SUVmax is depicted as M and SUVmean as Av.

For quantitative measurements, ^18^F-FDG PET/CT scans were analysed using dedicated software (OsiriX64-bit; OsiriX Imaging Software, Geneva, Switzerland). Two independent observers measured the SUV in five regions of interest (ROIs) at each aortic segment (ascending, arch and descending). These were performed sequentially along the length of the aorta every 5 mm on the axial slices. The maximum and mean standard uptake values were measured. The measurements were performed at a level distal to the origin of coronary vessels to avoid myocardial spillover. (Figure 2) For qualitative evaluation, the images were reviewed on an Advanced Window GE workstation using the CardIQ Xpress application. Vascular inflammation was assessed by the American Society of Nuclear Cardiologists visual grading criteria as follows: Grade 0, No vascular uptake (≤mediastinum); Grade 1, Vascular uptake < liver uptake; Grade 2, Vascular uptake = liver uptake, may be PET-positive; Grade 3, Vascular uptake > liver uptake, considered PET-positive (15). Vascular inflammation was determined to be present in patients with Grade 2 or Grade 3 uptake.

### Data analysis

In statistical analysis, continuous data was presented using the mean ± standard deviation (SD), while categorical data was presented using proportions. Interobserver variability between two observers was evaluated using several statistical methods to ensure a comprehensive understanding of agreement. The Bland-Altman method was employed to assess the agreement between observers. This technique involves plotting the differences between the two observers’ measurements against their averages, allowing for the identification of any systematic biases in measurements. Cohen’s Kappa Statistics were used to determine interobserver reliability; Cohen’s Kappa statistics were calculated. The interpretation of Kappa values is as follows: 0.1–0.2, slight agreement; 0.21–0.4, fair agreement; 0.41–0.6, moderate agreement; 0.61–0.8, substantial agreement; 0.81–1.0, almost perfect agreement. This metric provides a clear understanding of how much agreement exists beyond chance. Intraclass correlation coefficient (ICC) was also calculated to gauge interobserver reliability, with interpretations as follows: <0.5, poor reliability; 0.5–0.75, moderate reliability; 0.75–0.9, good reliability; >0.9, excellent reliability. Linear mixed-effects analysis was performed to account for the clustered structure of the data, with subjects treated as a random effect. This approach helps assess variability by separating subject variance from residuals, presenting both along with their standard deviations. All analyses were conducted using SPSS version 23 (SPSS Inc., Chicago, IL, USA), with statistical significance defined as p < 0.05 (16).

## Results

### Patient characteristics

Of the 64 consecutive patients identified with acute COVID-19, 33 were recruited, and 31 (94%) were black men from Kenya. Five active controls were recruited. A total of 30 patients had Cardiac ^18^F-FDG PET/CT data available for interobserver variability assessment (Figure 1). The median (IQR) age was 51 (34.57) years (interquartile range, IQR: 34–55). Patients were clinically stable and asymptomatic when imaged. Results showed that 63.3% had no inflammatory cell infiltration on Cardiac PET, 26% had type II diabetes, and 30% had systemic hypertension. They had an average of four days of COVID-19 symptoms prior to imaging and 50% required oxygen (Table 1).

**Table 1:** Baseline Characteristics of Patients With Acute COVID-19 (N = 33).


	PATIENTS

Demographics and past medical history	

Age, y	51 [34–56]

Current/exsmokers	6 (18.2)

Diabetes	10 (31)

Hypertension	11 (33)

HIV	4 (13)

Clinical assessments	

Symptom duration, days	4 [2–7]

Systolic BP, mm Hg	127 [120–136]

Diastolic BP, mm Hg	78 [70–85]

Heart rate, bpm	88 [80–92]

COVID-19 treatments	

Oxygen requirement	19 (58)

Remdesevir	4 (13)

Dexamethasone	15 (47)

Laboratory measurements	

Creatinine, μmol/L	97 [60–108]

C-reactive protein, mg/L	55 [25–101]

NT-proBNP, pg/mL	35 [28–151]

Troponin, ng/L	3.88 [2.76–7.18]


Data are provided as number (percentage) or median [interquartile range]. BP indicates blood pressure; NT-proBNP, N-terminal pro–brain natriuretic peptide; and PCR, polymerase chain reaction.

### Qualitative assessment

Agreement between the observers was evaluated qualitatively between observers x and y and for all measurements using Kappa level of agreement. There was excellent agreement between observers x and y for the aa and arch regions, with the Kappa coefficients for the interobserver agreement of 0.92 (95%CI: 0.78–1.0) and 0.91 (95%CI: 0.74–1.0), respectively. As for the da region, there was an acceptable level of agreement between observers x and y, with Kappa coefficient for the interobserver agreement of 0.79 (95%CI: 0.56–0.99) (Tables 2, 3 and 4).

**Table 2:** Kappa measures of agreements for the Ascending Aorta.


		OBSERVER Y		TOTAL

		ABSENT	PRESENT	

Observer X	Absent	20	0	20

	Present	1	9	10

	Total	21	9	30


Kappa: 0.92 (95%CI:0.78–1.0).

**Table 3:** Kappa measures of agreements for the Aortic Arch.


		OBSERVER Y		TOTAL

		ABSENT	PRESENT	

Observer X	Absent	11	1	18

	Present	2	10	12

	Total	19	11	30


Kappa: 0.79 (95%CI:0.56–1.0).

**Table 4:** Kappa measures of agreements for the Descending Aorta.


		OBSERVER Y		TOTAL

		ABSENT	PRESENT	

Observer X	Absent	22	0	22

	Present	1	7	8

	Total	23	7	30


Kappa: 0.91 (95%CI:0.74–1.0).

### Bland-Altman plots

#### 1. Mean standardized uptake values (SUVmean)

**Figure 3:**
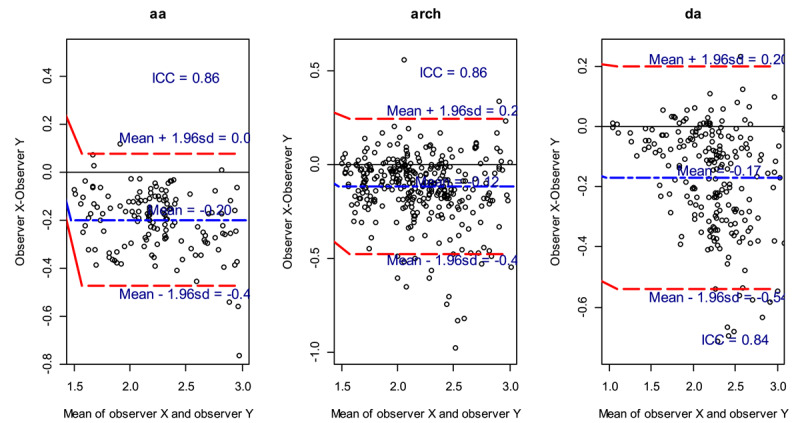
Mean Standardized uptake values (SUVmean) for different regions.

The plots show the average of the difference (bias) between the two observers (long dash blue line). The SUVmean bias and limits of agreement (LOA) were calculated as –0.20 and –0.47–0.08, –0.12 and –0.48–0.24 and –0.17 and –0.54–0.20 for the regions aa, arch and da, respectively (Figure 3). The negative bias (mean) for all the region suggested that observer y gave higher measurements compared to observer x for all the regions. The ICC showed good reliability between the two observers for all the regions measured (Figure 3). For mean regions aa, arch and da, the concordance were Kappa = 0.86 (95%CI: 0.24–0.95), Kappa = 0.86 (95%CI: 0.72–0.92) and Kappa = 0.85 (95%CI: 0.53–0.93), respectively, indicating perfect agreement between the observers (Table 5).

#### 2. Maximum standardized uptake values (SUVmax)

**Figure 4:**
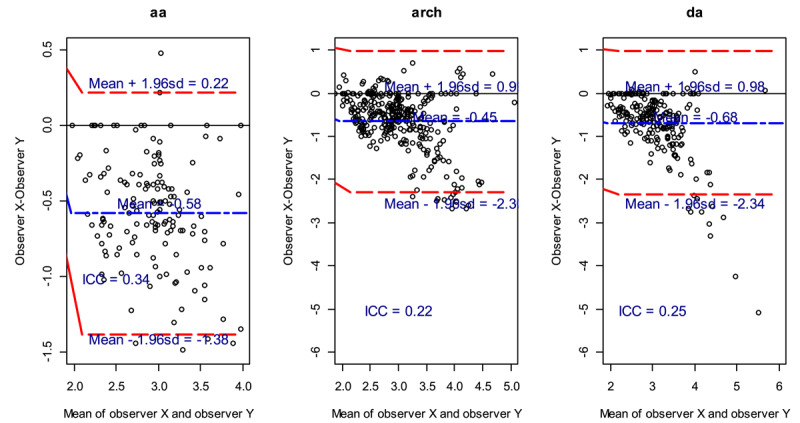
Maximum Standardized uptake values (SUVmax) for different regions.

The plots show the average of the difference (bias) between the two observers (long dash blue line). The bias and LOA for SUVmax regions were –0.58 and –1.38–0.22, –0.45 and –2.30–0.99 and –0.68 and –2.34–0.98 for the respective regions of aa, arch and da (Figure 4). The negative bias in all the cases indicates that observer y gave higher measurements compared to observer x for all the regions. The ICC showed poor reliability between the two observers (Figure 4). For the SUVmax regions, the concordance were Kappa = 0.46 (95%CI: –0.037–0.71), Kappa = 0.32 (95%CI: 0.091–0.49) and Kappa = 0.34 (95%CI: 0.087–0.53) for aa, arch and da regions, respectively. The concordance for SUVmax region showed a moderate level of agreement in aa, while there was fair agreement in the other regions (Table 5).

### Kappa measures of agreements

Agreement between the observers was also evaluated qualitatively between the first observer and that of the second observer for all measurements using Kappa level of agreement. There was an excellent agreement between observer 1 and observer 2 for aa and arch regions, with Kappa coefficient for the interobserver agreement of 0.92 (95%CI: 0.78–0.99) and 0.91 (95%CI: 0.74–0.99), respectively. As for the da region, there was an acceptable level of agreement between observers 1 and 2, with Kappa coefficient for the interobserver agreement of 0.79 (95%CI: 0.56–0.99) (Table 5).

**Table 5:** Kappa agreement for SUVmean and SUVmax with corresponding 95%CI.


REGION	SUVMEAN		SUVMAX		ALL	
		
	KAPPA	95%CI	KAPPA	95%CI	KAPPA	95%CI

Aa	0.86	0.24–0.95	0.46	0.037–0.71	0.92	0.78–0.99

Arch	0.86	0.72–0.92	0.32	0.091–0.49	0.79	0.56–0.99

Da	0.85	0.53–0.93	0.34	0.087–0.53	0.91	0.74–0.99


### Linear mixed effects models

Table 6 presents variance estimates from a linear mixed-effects model for the outcome variables ‘SUV mean (Y)’ and ‘SUV max (Y),’ stratified by three regions (aa, arch and da). The SUVmax outcome shows higher subject-to-subject variability than SUVmean across all regions, indicating greater variability in the maximum value of SUV across regions. For the arch and da regions, both the variance and residuals for SUV max are higher as compared to the aa region.

**Table 6:** Linear mixed effects model estimates for variance and residuals across all the regions.


REGION	SUV MEAN		SUV MAX	
	
	SUBJECT VARIANCE (SD)	RESIDUAL (SD)	SUBJECT VARIANCE (SD)	RESIDUAL (SD)

Ascending aorta	0.011 (0.0105)	0.009 (0.094)	0.089 (0.299)	0.072 (0.268)

Aortic arch	0.102 (0.319)	0.013 (0.114)	0.249 (0.500)	0.448 (0.669)

Descending aorta	0.033 (0.182)	0.013 (0.116)	0.519 (0.721)	0.210 (0.459)


## Discussion

The use of a visual grading scale (vascular to liver uptake) is the gold standard endorsed by the joint collaboration of the EACVI (European Association of Cardiovascular Imaging) and the EANM (European Association of Nuclear Medicine) on procedural recommendations of cardiac PET/CT imaging (17). This study demonstrates excellent interobserver correlation for visual grading of the ^18^F-FDG PET uptake for the aa, arch, and da. The Kappa coefficients in these regions were 0.86 (95%CI: 0.24–0.95), 0.86(95% CI: 0.72–0.92) and 0.85 (95%CI: 0.53–0.93), respectively. These findings are similar to those of Lensen et al., who, in a LVV, found an interobserver agreement between Kappa: 0.96 and 0.79 when vascular wall ^18^F-FDG uptake higher than liver uptake was used as a diagnostic criterion (8).

The guidelines also recommend quantification of ^18^F-FDG PET activity, using SUVmax and SUVmean (17). The quantitative SUVmean in this study showed a very high positive correlation between the two observers; aa was r = 0.96 (p < 0.001), arch was r = 0.87 (p < 0.001) and da was r = 0.89 (p < 0.001) with narrow confidence intervals. The intraclass correlation for the agreement of SUVmean measurements between the two observers indicated perfect agreement. The SUVmean gives an average of all voxels in the ROI, and thus, in this study, it showed the highest level of reproducibility.

The SUVmax correlation between observers were substantially strong for the three aortic anatomical regions. In the aa region: r = 0.75, p < 0.00, arch region: r = 0.64, p < 0.001 and da region: r = 0.70, p < 0.001. The confidence intervals for the SUVmax were wider compared to the SUVmean, hence the relationship between observers x and y is stronger in the SUVmean compared to the SUVmax. Despite the negative bias for the SUVmean demonstrating that observer y gave higher measurements compared to observer x for all the regions, the intraclass correlation for the agreement measurements between the two observers showed the concordance were Kappa = 0.86 (95%CI: 0.24–0.95), Kappa = 0.86 (95%CI: 0.72–0.92) and Kappa = 0.85 (95%CI: 0.53–0.93), respectively, indicating perfect agreement between the observers. The SUVmax interobserver variability in all aortic regions showed a substantial agreement.

Therefore, this study showed that the interobserver agreement was excellent for SUVmean in all regions and substantial for SUVmax. This concurs with a study done by Irene et al. that found repeatability of SUVmean is superior to SUVmax in ^18^F-FDG quantification in tumor imaging (18). Interobserver variability of up to 17% for some SUVmean has been noted, whereas interobserver variability in determining change in SUVmax of 16.7% ± 36.2% has been found due to different ROI placements. (9) Thus, the quantitative characteristics of ^18^F-FDG PET are increasingly recognized as providing a more accurate and less observer-dependent measure of inflammatory atherosclerosis (19). SUVmax is regularly used for quantification in PET-atherosclerosis studies (20).

The findings of moderate reproducibility of SUVmax are not as robust as the findings of Rudd et al., who showed that maximum TBR measurements for quantifying ^18^F-FDG uptake in atherosclerotic vascular imaging were highly reproducible (21). Karel-jan et al., in a study on variability in quantitative analysis of atherosclerotic plaque inflammation using ^18^F-FDG PET/CT by evaluation of SUVmax of ROIs, found interobserver agreement was excellent for all vascular segments, and more so in aortic segments (22).

The moderate interobserver agreement for SUVmax may be explained by the inclusion of areas of high ^18^F-FDG uptake outside of the aortic wall. In particular, our study population had active COVID-19 infection, and a majority of the patients had ^18^F-FDG avid reactive mediastinal and hilar lymph nodes, which may have been erroneously included in the ROI of the vascular wall measurement. The SUVmax picks the highest voxel value within the ROI, but in small ROIs, it is more susceptible to noise from peri-aortic structures such as fat, nodes and renal structures. Thus, this may account for the sub-excellent interobserver variability as compared to patients with atherosclerotic aortic lesions.

The limitations of this study were the small sample size of patients included in the study, which affects the generalizability of the findings. However, each observer had to make at least 15 measurements for each subject for the quantitative analysis and thus can compensate for the small population. In addition, the study population has an active inflammatory process due to COVID-19 infection, hence the finding of multiple mediastinal and para-aortic nodes with avid ^18^F-FDG uptake. This can account for the seemingly wider interobserver variability of the SUVmax readings as compared to similar studies which mainly studied population with atherosclerotic lesions and non-infectious inflammatory vasculitis. There were only two observers in this study.

## Conclusion

This study shows an excellent interobserver reproducibility for both qualitative and quantitative SUVmean vascular ^18^F-FDG measurements in patients with COVID-19 LVV. Quantitative SUVmax demonstrated moderate interobserver agreement possibly due to avid para-aortic node uptake and interference with the measurements.

## Additional File

The additional file for this article can be found as follows:

10.5334/gh.1433.s1 Supplementary materials. Datasets.
